# CytoSimplex: visualizing single-cell fates and transitions on a simplex

**DOI:** 10.1093/bioinformatics/btaf119

**Published:** 2025-03-22

**Authors:** Jialin Liu, Yichen Wang, Chen Li, Yichen Gu, Noriaki Ono, Joshua Welch

**Affiliations:** Department of Computational Medicine and Bioinformatics, University of Michigan, Ann Arbor, MI, 48109, United States; Department of Computational Medicine and Bioinformatics, University of Michigan, Ann Arbor, MI, 48109, United States; Department of Computational Medicine and Bioinformatics, University of Michigan, Ann Arbor, MI, 48109, United States; Department of Electrical and Computer Engineering, University of Michigan, Ann Arbor, MI, 48109, United States; University of Texas Health Science Center at Houston School of Dentistry, Houston, TX, 77030, United States; Department of Computational Medicine and Bioinformatics, University of Michigan, Ann Arbor, MI, 48109, United States; Department of Computer Science and Engineering, University of Michigan, Ann Arbor, MI, 48109, United States

## Abstract

**Summary:**

Cells differentiate to their final fates along unique trajectories, often involving multi-potent progenitors that can produce multiple terminally differentiated cell types. Recent developments in single-cell transcriptomic and epigenomic measurement provide tremendous opportunities for mapping these trajectories. The visualization of single-cell data often relies on dimension reduction methods such as UMAP to simplify high-dimensional single-cell data down to an understandable 2D form. However, these dimension reduction methods are not constructed to allow direct interpretation of the reduced dimensions in terms of cell differentiation. To address these limitations, we developed a new approach that places each cell from a single-cell dataset within a simplex whose vertices correspond to terminally differentiated cell types. Our approach can quantify and visualize current cell fate commitment and future cell potential. We developed CytoSimplex, a standalone open-source package implemented in R and Python that provides simple and intuitive visualizations of cell differentiation in 2D ternary and 3D quaternary plots. We believe that CytoSimplex can help researchers gain a better understanding of cell type transitions in specific tissues and characterize developmental processes.

**Availability and implementation:**

The R version of CytoSimplex is available on Github at https://github.com/welch-lab/CytoSimplex. The Python version of CytoSimplex is available on Github at https://github.com/welch-lab/pyCytoSimplex.

## 1 Introduction

The key goal of CytoSimplex is to quantify and visualize the current state and future differentiation potential of cells undergoing fate transition. Before cells reach their final fates, they often pass through intermediate multipotent states where they have characteristics and potential to generate multiple lineages. Single-cell RNA (scRNA) and epigenome sequencing methods provide temporal snapshots of cells at different moments during cell differentiation. However, since each cell is seen only once and cannot be followed longitudinally, computational approaches must be used to infer where each cell is on its journey. Pseudotime inference and RNA velocity methods have both been used extensively for this task (Ji and Ji 2016, Welch *et al.* 2016, La Manno *et al.* 2018, Street *et al.* 2018, Cao *et al.* 2019, Saelens *et al.* 2019, Setty *et al.* 2019, Bergen *et al.* 2020, Gu *et al.* 2022, Li *et al.* 2023). RNA velocity in particular has gained popularity recently. However, the results of these inference methods are often visualized using 2D projections of the data derived from principal components, t-distributed Stochastic Neighbor Embeddings, and Uniform Manifold Approximation and Projection (UMAP). While these can be useful visualization tools, they inevitably distort the distance relationships among cells when reducing high-dimensional data to two dimensions (Gorin *et al.* 2022). The problem is particularly acute when displaying pseudotime or RNA velocity results; the 2D coordinates are not optimized to show the inferred developmental relationships (Atta *et al.* 2022) and can thus lead to incorrect conclusions about the fate similarity and future potential of multipotent cells. On the other hand, previous computational tools including archetype analysis (Hart *et al.* 2015, Korem *et al.* 2015, Nath *et al.* 2021, Groves *et al.* 2022) allow the comparison of similarities between cells and archetypal cells based on a similar concept of simplex. However, these methods do not incorporate predicted future states of cells. Moreover, they can only be applied to scRNA-seq data but not other protocols including single-nucleus ATAC (snATAC) and multi-omic data. Existing methods such as CellRank (Lange *et al.* 2022) that can compute fate probabilities use more complicated algorithms under the hood which weaken the models’ interpretability and can cause unexpected differences from velocity results.

To address these challenges, we developed a new visualization method called CytoSimplex. The key idea of CytoSimplex is to choose clearly defined, interpretable landmarks and plot the positions of transitional cells relative to them. Specifically, we use the molecular signatures of final fates as the vertices of a simplex, and then calculate the position of each cell within the simplex. This makes it easier to avoid misleading interpretations due to unclear relationships among cell types on UMAP plots.

## 2 Methods

In CytoSimplex, we model the space of lineage differentiation as a simplex with vertices representing potential terminal fates. A simplex extends the idea of a triangle into any dimension; where a point is a 0D simplex, a line segment is a 1D simplex, a triangle is a 2D simplex, and a tetrahedron is a 3D simplex. A simplex often represents variables that add up to 1 or another fixed value. Intuitively, this is an excellent model for representing cell fate commitment, because there is a small number of cell fates that a given progenitor can produce. This constant sum means the variables cannot change independently, resulting in K-1 degrees of freedom for a K-dimensional simplex. Therefore, a 3-simplex, having two degrees of freedom, can be charted as a 2D triangle. Previously, simplex plots have been used in chemistry, geology, ecology, and other scientific fields (Aitchison 1982, Twarakavi *et al.* 2010, Berndt and Klemme 2022, Cooney *et al.* 2022). Ternary plotting has also been used in some single-cell analyses to demonstrate cell-type proportions (Pal *et al.* 2021).

**Figure 1.**
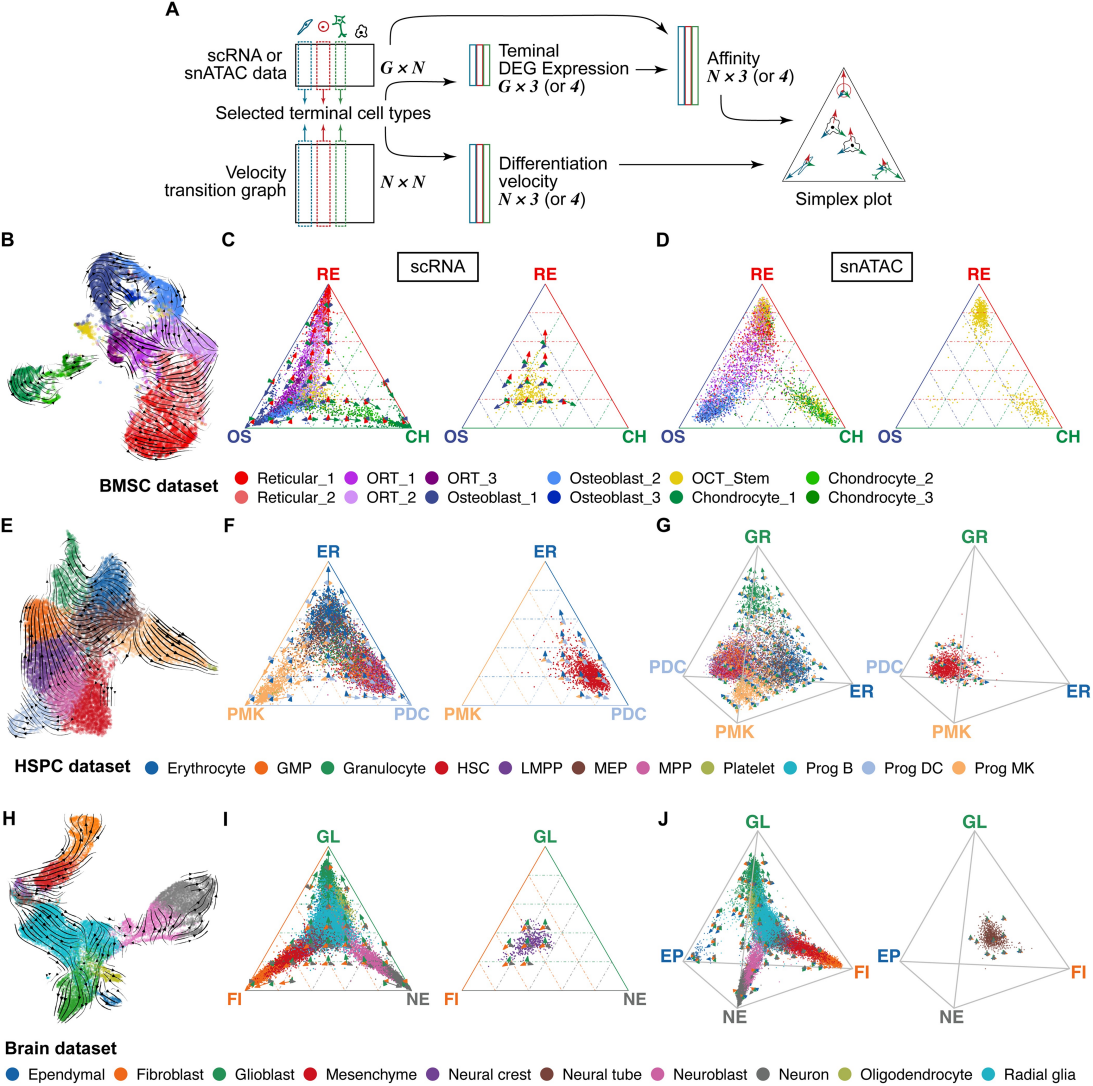
CytoSimplex shows multi-lineage differentiation potential of progenitor cells in different organs. (A) Input and output of CytoSimplex. CytoSimplex takes a gene-by-cell RNA and/or ATAC count matrix, RNA velocity transition matrix, and cluster annotations as input, and generates ternary or quaternary plots. The coordinates of each cell within the simplex analysis indicate the cell’s differentiation potential toward each terminal fate. The color coding of both the arrows and vertices corresponds to the final fates selected. Together, these simplexes and arrows provide an intuitive visualization of cellular trajectories. (B) RNA velocity of major cell types in mouse bone marrow. The velocity streamline plot is generated by scVelo. The streamlines connect cells on a differentiation path together and arrows indicate path directions. Corresponding to panel (B) and (C), RNA dynamic vectors originate from OCT-stem cells and point to other differentiated cell types, suggesting OCT-stem cells as the cell-of-origin cluster. (C, D) Ternary plots of representative cell types in embryonic mouse bone marrow generated from scRNA and snATAC data. OCT stem cells demonstrate transcriptomic potential toward all three terminal fates, while also showing epigenomic potential toward Reticular and chondrocyte cells. Red arrow and axis: Reticular cluster (RE). Blue: Osteoblast cluster (OS). Green: Chondrocyte cluster (CH). (E) RNA velocity of major cell types in HSPC data. HSC and MEP, functioning as progenitor cell types, serve as two distinct cell-of-origin clusters. (F, G) Ternary and quaternary plots of human hematopoietic stem and progenitor cells (HSPC). Corresponding to their locations on the UMAP plots in (E), progenitor cell types including HSC and MEP show distinct transcriptomic similarities to all terminal fates, while showing identical differentiation potential toward all vertices, regardless of in the ternary or quaternary plot. HSC: Human hematopoietic stem, MEP: Megakaryocyte-erythrocyte progenitor. Blue arrow and axis: Erythrocyte cluster (ER). Yellow: Progenitor Megakaryocyte cluster (PMK). Light blue: Progenitor Dendritic cluster (PDC). Green: Granulocyte cluster (GR). (H) RNA velocity of major cell types in mouse brain atlas. Neural tube and neural crest cell serve as root cells of distinct differentiation trajectories while also sharing a similar location in UMAP plot. (I, J) Ternary and quaternary plots of major cell types in mouse brain atlas. Neural tube and neural crest cell, as two topologically close stem cell types, share similar transcriptomic similarities and differentiation potential toward all fates in ternary and quaternary plots. Green arrow and axis: Glioblast (GL). Orange: Fibroblasts (FI). Gray: Neurons (NE). Blue: Ependymal cells (EP).

We represent the molecular state of a cell as a point located within the simplex. The coordinates of each point (cell) represent its affinity toward each vertex, with these affinities summing to unity. In addition, we incorporate arrows to denote the RNA velocity of each cell toward each terminal state. These simplexes and arrows together serve as cell-wise representations of putative transcriptomic and epigenomic transitions toward a specific trajectory, guided by the selection of putative terminal fates. CytoSimplex takes scRNA and/or single-cell ATAC count matrices, a cell-cell transition graph [such as a result from RNA velocity analysis (La Manno *et al.* 2018, Bergen *et al.* 2020)], and cell type annotations as input (Fig. 1A, left). To generate simplex dot plots, for each dataset with G features and C cells and N chosen terminal cell types, we first derive a distance metric by selecting the top 30 differentially expressed marker genes by Wilcoxon test. Alternatively, we provide Principal Component Analysis (PCA) as another option. Next, we locate the mean-centroids of the terminal cell types, denoted as $V^{G\times N}$, and calculate the Euclidean distance ${D^{C\times N}}_{\geq 0}$ from each cell in each dataset $E^{G\times C}$ to V.


(1) $$D_{c,n}=\sqrt{\sum_{i=1}^{G}{\left(E_{i,c}-V_{i,n}\right)}^{2}}$$

The distances from each cell to N selected terminals are normalized to unity. We then apply a Gaussian kernel to convert the distances to similarities, denoted as ${S^{C\times N}}_{>0\;\mathrm{and}\;\leq 1}$.


(2) $$S_{c,n}=e^{-\frac{D_{c,n}}{\left\|D_{c,}\right\|\sigma }}$$

The similarities to each terminal are scaled by the range, resulting in ${S^{\left(s\right)}}_{c,n}$, where $\min(S_{,n})$ and $\max(S_{,n})$ denotes the minimum and maximum value of nth column of S, respectively.


(3) $$S_{c,n}^{\left(s\right)}=\frac{S_{c,n}-\min(S_{,n})}{\max\left(S_{,n}\right)-\min(S_{,n})}$$

The scaled similarity for each cell is L1-normalized to address the unity sum.


(4) $${\hat{S}}_{c,n}=\frac{S_{c,n}^{\left(s\right)}}{\left\|S_{c,}^{\left(s\right)}\right\|}$$

This is the barycentric coordinate that is finally used for projection to a simplex. To visualize velocity vectors, we first derived a cell-cell transition graph with an RNA velocity method such as MultiVelo (Li *et al.* 2023), VeloVAE (Gu *et al.* 2022), or scVelo (La Manno *et al.* 2018). We then calculated the future differentiation potential of each cell by taking the average of the edge weights between each cell and the cells in each terminal cell type. For a concise view, we binned neighboring cells projected on the simplex embeddings into square or cube grids, aggregated the weights by the total number of cells in each grid, and normalized these grid weights to a sum of unity. Finally, the velocity was depicted as arrows pointing from the centroid of each grid to each vertex, with arrow length representing the normalized weights (Fig. 1A, right).

CytoSimplex is different from prior methods due to its novelty of showing both the current and future state of each cell. Previous methods like archetype analysis (Hart *et al.* 2015, Korem *et al.* 2015, Nath *et al.* 2021, Groves *et al.* 2022)—which resembles the simplex concept—allow the comparison of similarities between cells and terminal cell types in a low-dimensional space defined by archetypal cells, and therefore help determine if cells are multitasking during development. Yet CytoSimplex not only shows the relationships among current cell states, but also the fate potential and predicted future states of the cells. This unique feature allows CytoSimplex to accurately represent cell lineage transitions dynamically and intuitively, measuring how much potential each cell has for differentiating into each terminal fate.

CytoSimplex is versatile and compatible with multiple sequencing protocols. Our method takes any cell-by-feature matrices accompanied by cell-cell transition graphs and cell-type annotations as input. We emphasize that CytoSimplex is a visualization method with the functionality to incorporate a quantitative cell-cell transition matrix inferred by RNA velocity to visualize cell transition trajectories. Therefore, showing potential uncertainty of RNA velocity inference is beyond the scope of our tool. Additionally, although it is possible in principle to visualize a simplex with >4 vertices, our tool does not currently support this. Thus, we recommend using CytoSimplex to visualize only 2–4 fates at once, subsetting if needed in more complex datasets.

To enhance user-friendliness, we have developed an open-source package in both R and Python. This package is carefully tailored for researchers who are not familiar with programming. We aim to contribute to the bioinformatics community by distributing our package to the widest range of researchers.

## 3 Results

### 3.1 CytoSimplex identifies single-cell cell fate transitions in the mouse bone marrow

To illustrate how CytoSimplex can be used, we first used our method on mouse bone marrow stromal cells (BMSCs) scRNA-seq and snATAC-seq data from our recent papers (Welch *et al.* 2019, Liu *et al.* 2020, Matsushita *et al.* 2020, Matsushita *et al.* 2023). This BMSC population encompasses large groups of osteoblastic cells (Osteoblasts, OS), pre-adipocyte-like reticular cells (Reticular cells, RE), separate clusters of chondrocytes (Chondrocytes, CH), and a putative stem cell cluster with osteoblast-chondrocyte transitional identities (OCT stem cells) (Fig. 1B). We applied CytoSimplex on the velocity outputs from scVelo (La Manno *et al.* 2018). Velocity analysis suggests that OCT stem cells provided a robust cellular origin of osteoblasts, reticular cells, and their intermediate-state cells (Fig. 1B). CytoSimplex confirms but deepens the story by demonstrating the transcriptomic similarity and differentiation potential of each cell in the dataset. Specifically, in our simplex plots, the majority of OCT stem cells stay in the middle of the simplex and have almost equal similarity to each fate, while also demonstrating “trilineage” potential toward all three fates indicated by arrows pointing toward three vertices (Fig. 1C, right). In contrast, the chromatin accessibility profiles of OCT stem cells reveal “trilineage” potential that aligns with the transcriptomic profiles, but with a greater bias toward the reticular and chondrocyte fate (Fig. 1D, right). In comparison, cells within each terminal cluster exhibited strong RNA and ATAC similarities to their respective fates (Fig. 1C and D, Supplementary Figs S2 and S3). Therefore, CytoSimplex cannot only detect the multipotency of a potential stem cell population, but also uniquely visualize differences between the transcriptomic and epigenomic developmental direction of OCT-stem cells.

### 3.2 CytoSimplex demonstrates single-cell fates in the human hematopoietic stem and progenitor cells

Subsequently, we applied our method to human hematopoietic stem and progenitor cells (HSPCs) single-nucleus RNA+ATAC multiome data used in our published study (Li *et al.* 2023). Our goal in analyzing this dataset is not to discover new properties of HSPC differentiation, but to demonstrate how our approach allows visualizing known biology. This dataset comprises two distinct global populations of stem cells, namely the hematopoietic stem cells (HSCs) and multipotent progenitors (MPPs). These cells are capable of differentiating into three main paths that connect megakaryocyte/erythroid/mast cell lineages (MEMPs), granulocyte progenitors (GPs), and lymphoid-myeloid progenitors (LMPs) (Ranzoni *et al.* 2021). Each lineage consists of various progenitor cell types including megakaryocyte-erythroid progenitors (MEPs), as well as more differentiated blood cells such as granulocytes (GRs), erythrocytes (ERs), and platelets. To demonstrate CytoSimplex’s capabilities, we picked three more differentiated fates on the paths of MEMPs and LMPs as the vertices, including progenitor dendritic cells (pDCs), megakaryocyte-erythrocyte progenitors (MEPs), and erythrocytes (ERs). Our ternary plots show that HSCs have a potential proximity with progenitor dendritic cells (pDCs), while MEP cells exhibit similarities to the ER and PMK fates in the transcriptional space. The velocity and cell-transition results from MultiVelo (Li *et al.* 2023) were taken as the input of CytoSimplex. This is consistent with our previous RNA velocity streamline plot based on UMAP (Fig. 1E). However, CytoSimplex also highlights the divergence between HSC and MEP cells. HSCs exhibit high differentiation potential across all vertices, while MEP cells tend to favor the ER and PMK vertices (Fig. 1F and G, right, Supplementary Fig. S4). Furthermore, the quaternary plots unveiled the same story when we included granulocytes (GRs), which are differentiated from granulocyte-monocyte progenitors (GMPs) instead of MEPs, as an additional vertex (Fig. 1G, Supplementary Fig. S1A). We also found that except for the ones serving as the vertices, none of the progenitor cell types reach the endpoints of any vertices in both ternary and quaternary plots (Supplementary Figs S4 and S5). This aligns with our biological expectation that the majority of differentiating progenitor cells are multipotent cells that have not yet reached their final fates. Therefore, our simplex method provides a clearer understanding of the intricate relationship between progenitor cells from multiple lineages and also highlights their pluripotent state.

### 3.3 CytoSimplex illustrates single-cell fates in the mouse brain

Moreover, we applied our method to part of a mouse brain atlas. In our previous work, we performed velocity analysis with VeloVAE (Gu *et al.* 2022) and demonstrated three distinct differentiation paths including glioblasts, fibroblasts, and neurons (La Manno *et al.* 2021, Gu *et al.* 2022). The progenitor cells, namely neural crest and neural tube cells, can develop into multiple descendants. In particular, neural crest cells develop into fibroblast cells via mesenchymal cells, while neural tube cells end up becoming both neuronal and non-neuronal cells, including oligodendrocytes, ependymal, and glioblast cells. While the velocity stream generally follows the direction of these differentiation paths, some streamlines can be misleading due to distorted cell-cell relationships introduced by dimension reduction (Gorin *et al.* 2022). For example, velocity arrows of neuroblast cells on the UMAP deviate largely from major paths from radial glial cells and toward neurons (Fig. 1H). In contrast, our ternary plot indicates an overall evident trend of neuroblast cells differentiating into neurons, with some cells showing similarities toward the glioblast fate (Fig. 1I, Supplementary Fig. S6). We further highlight that CytoSimplex captures high similarity in differentiation tendency between two topologically close stem cell types, the neural tube and neural crest cell, while still capturing the subtle distinctions. Specifically, neural crest cells have a higher tendency to undergo fibroblast cell transformation in comparison to neural tube cells (Supplementary Fig. S6). To demonstrate a four-lineage viewpoint, we introduced Ependymal as an additional end fate (Fig. 1J, Supplementary Fig. S1B). Progenitor cells, such as neural crest and neural tube cells, exhibit a similar neutral affinity toward all four vertices. CytoSimplex also validates clear differentiation trends indicated by RNA velocity streams, such as mesenchymal cells predominantly differentiated toward the fibroblast fate (Supplementary Fig. S6). Additionally, cells in each terminal cell type demonstrated the strongest similarities and differentiation trend toward their own fates (Supplementary Fig. S6).

## 4 Conclusion

In conclusion, CytoSimplex is an accessible and innovative tool that provides simple and intuitive visualization of complex cell transition trajectories. Its unique ternary or quaternary plots provide cell-wise information by combining cellular transcriptome or epigenomic signatures with RNA velocity-inferred differentiation potential, which allows researchers to perform high-resolution quantitative analyses compared to commonly used visualization methods. By utilizing CytoSimplex, researchers can easily focus on each lineage within a complex dataset and discern minor heterogeneities between intriguing cell populations, such as stem cells and progenitor cells. Furthermore, CytoSimplex’s implementation in R and python enables researchers to easily streamline their analysis with other single-cell analysis tools, improving their ability to analyze complicated datasets. We believe that CytoSimplex can greatly help advance the understanding of biological processes underlying cell differentiation.

## Supplementary Material

btaf119_Supplementary_Data
