# Supplementary material for: CytoSimplex: visualizing single-cell fates and transitions on a simplex
Source: Bioinformatics. 2025 Mar 22;41(4):btaf119. doi: 10.1093/bioinformatics/btaf119 (PMC11992338; doi:10.1093/bioinformatics/btaf119)
Supplement: btaf119_Supplementary_Data [file btaf119_supplementary_data.zip › Supplementary_Data.docx]

CytoSimplex: Visualizing Single-cell Fates and Transitions on a Simplex

Jialin Liu, Yichen Wang, Chen Li, Yichen Gu, Noriaki Ono, Joshua Welch

Supplementary Material

Table of Content

Supplementary Figure S1. Animated 3D quaternary simplex plots of HSPC and mouse brain atlas data. See separate file Supplementary_Figure_S1.gif.

- Supplementary Figure S2. Ternary simplex and velocity analyses of BMSC data.
- Supplementary Figure S3. Ternary simplex and velocity analyses of BMSC data.
- Supplementary Figure S4. Simplex and velocity analyses of HSPC data.
- Supplementary Figure S5. Simplex and velocity analyses of HSPC data.
- Supplementary Figure S6. Simplex and velocity analyses of mouse brain atlas data.

Supplementary Figure S7. Different approaches to visualize the cell fate of the BMSC dataset.

**Supplementary Figure S1. Animated 3D quaternary simplex plots of HSPC and mouse brain atlas data. See separate file Supplementary_Figure_1.gif.** Continuing from Figure 1, these animated quaternary simplex plots of HSPC (A) and mouse brain (B) data provide a full 360-degree view and therefore offer a more detailed demonstration of the relationship between selected cell types and terminal cell fates. For HSPC data, blue arrow and axis: Erythrocyte cluster (ER). Yellow: Progenitor Megakaryocyte cluster (PMK). Light blue: Progenitor Dendritic cluster (PDC). Green: Granulocyte cluster (GR). For mouse brain atlas data, green arrow and axis: Glioblast cluster (GL). Gray: Neuron cluster (NE). Orange: Fibroblast cluster (FI). Blue: Ependymal cluster (EP). Dot colors are indicated by the legends.


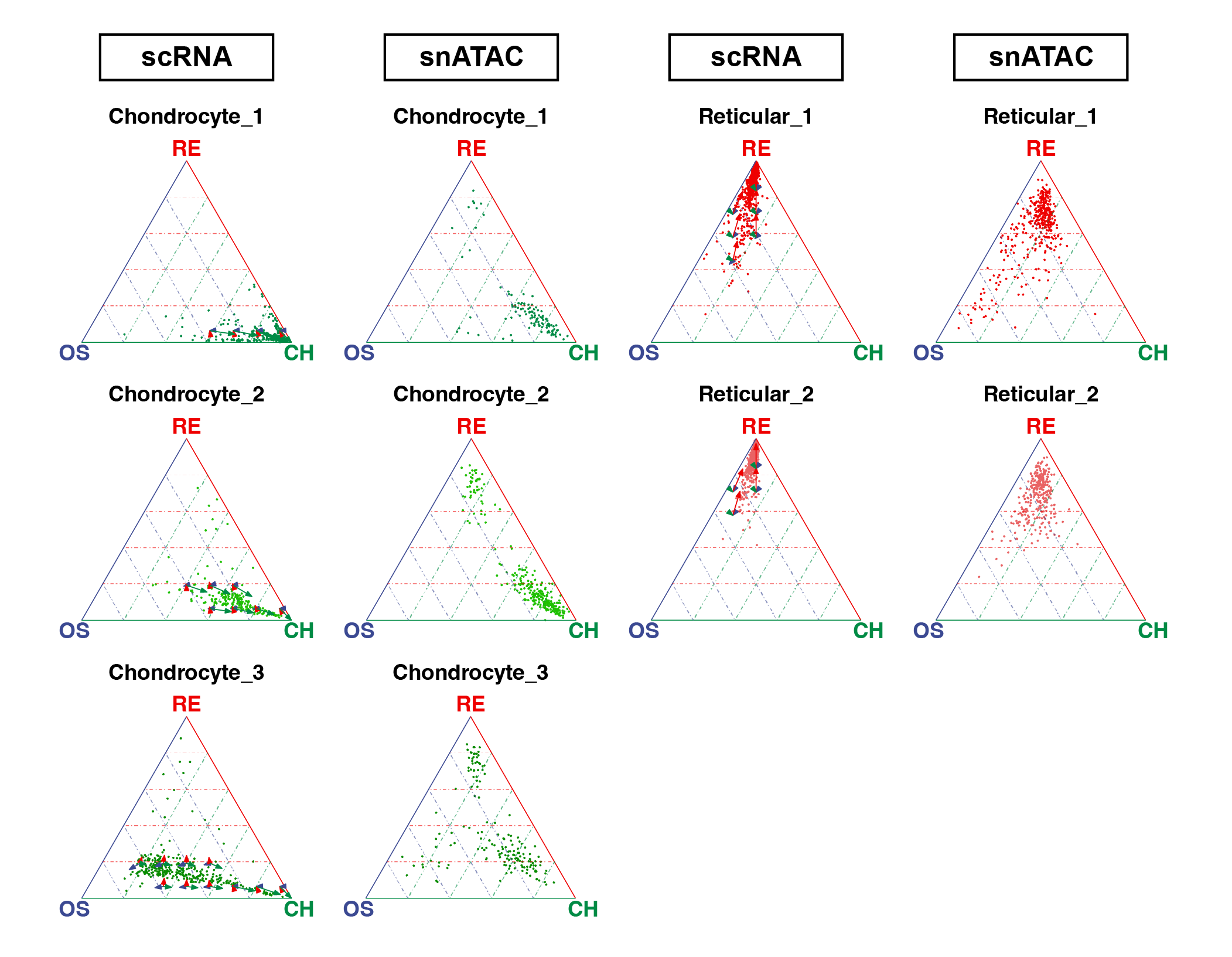
**Supplementary Figure S2. Ternary simplex and velocity analyses of BMSC data.** In the first and third columns, dots demonstrate the cell transcriptomic affinities towards three selected vertices, whereas the arrows show the future differentiation potential of the cells. In the second and fourth columns, dots demonstrate the cell epigenomic affinities towards three vertices. Red arrow and axis: Reticular cluster (RE). Blue: Osteoblast cluster (OS). Green: Chondrocyte cluster (CH). Dot colors indicate clusters.


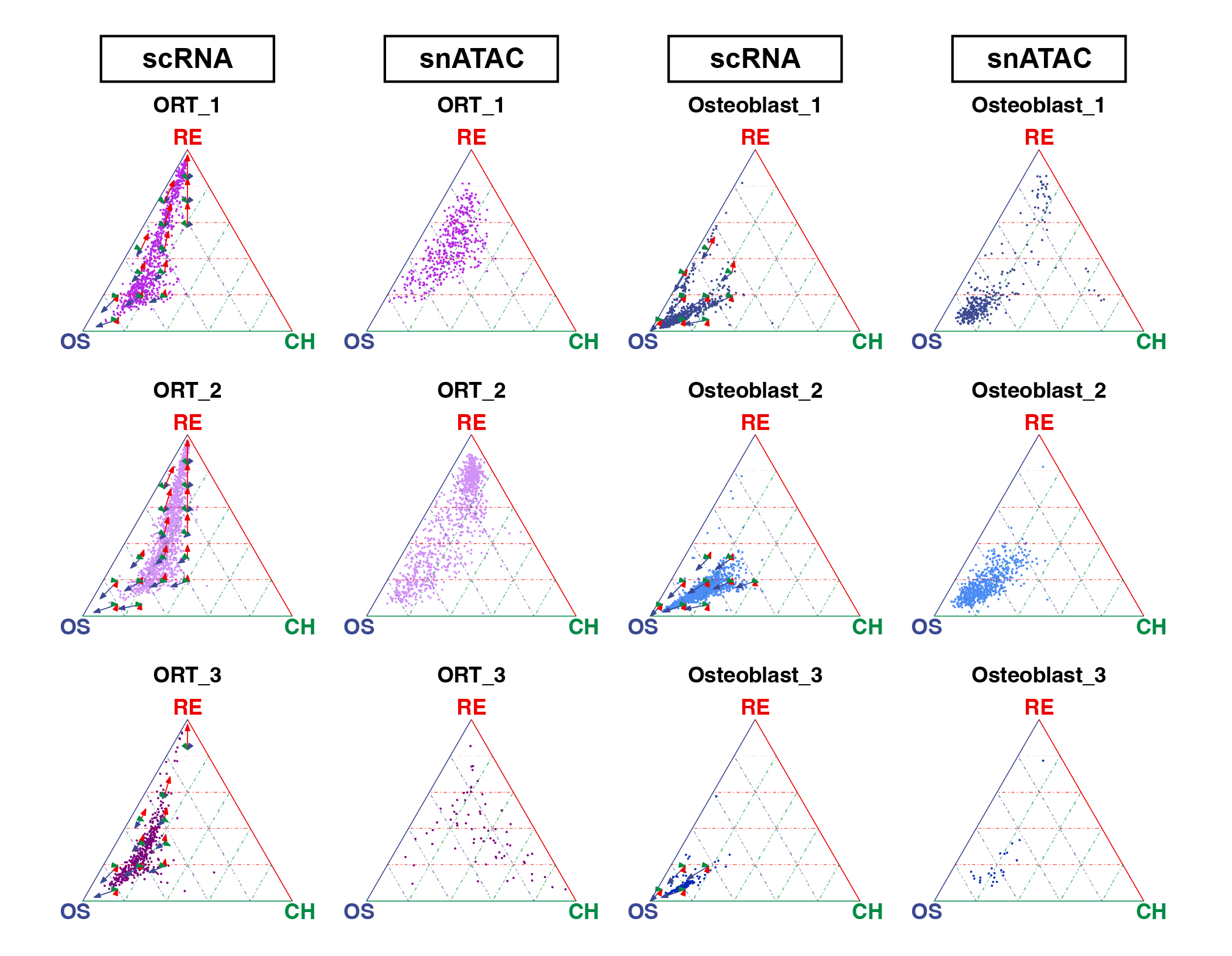
**Supplementary Figure S3. Ternary simplex and velocity analyses of BMSC data.** Continuing from **Supplementary Figure 2**, this figure includes the remaining clusters from BMSC data. Red arrow and axis: Reticular cluster (RE). Blue: Osteoblast cluster (OS). Green: Chondrocyte cluster (CH). Dot colors indicate clusters.


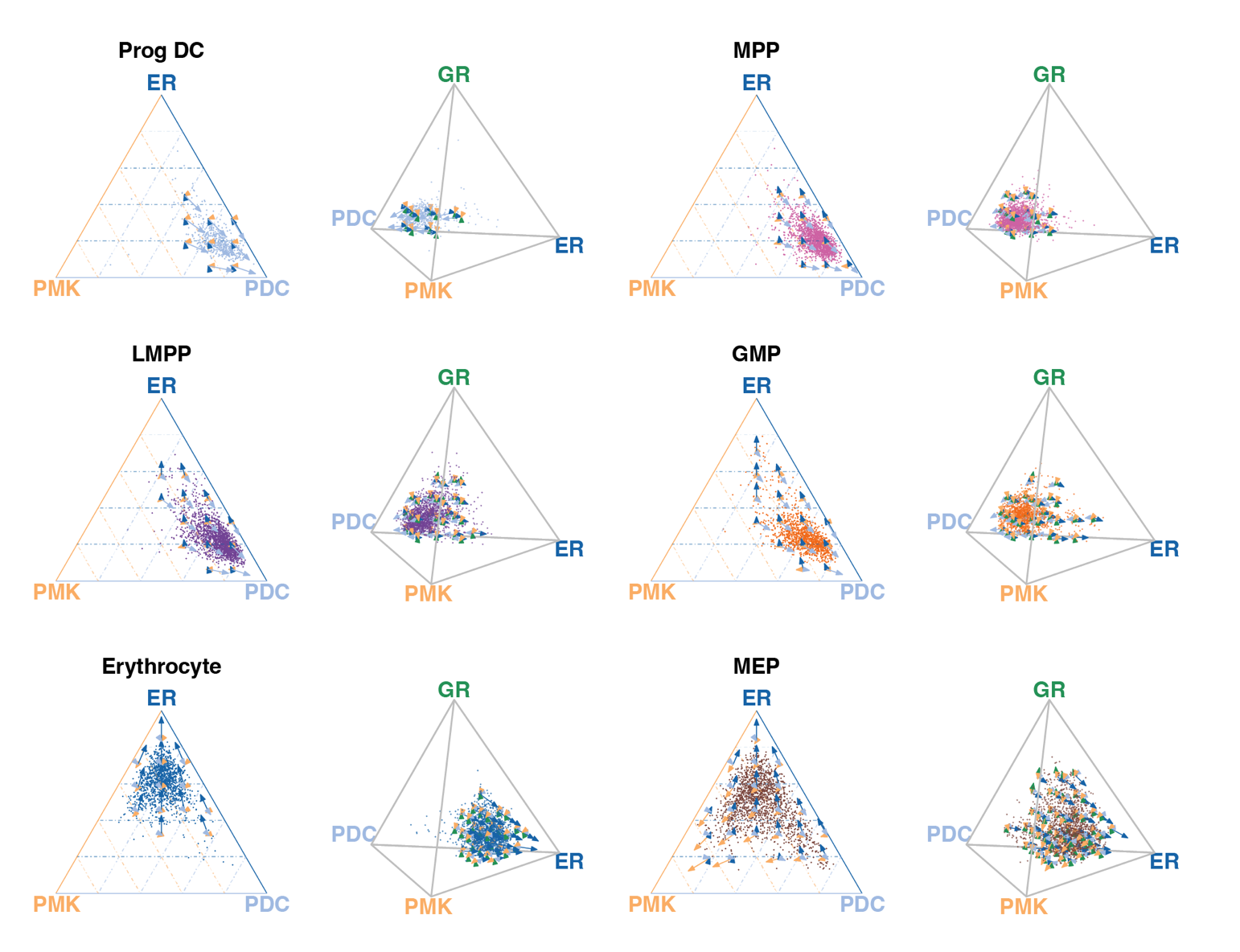
**Supplementary Figure S4. Simplex and velocity analyses of HSPC data.** Dots demonstrate the cell transcriptomic affinities towards three selected vertices, whereas the arrows show the future differentiation potential of the cells. Ternary simplex plots are shown in the first and third columns, while quaternary simplex plots are shown in the second and fourth columns. Blue arrow and axis: Erythrocyte cluster (ER). Yellow: Progenitor Megakaryocyte cluster (PMK). Light blue: Progenitor Dendritic cluster (PDC). Green: Granulocyte cluster (GR). Dot colors indicate clusters.


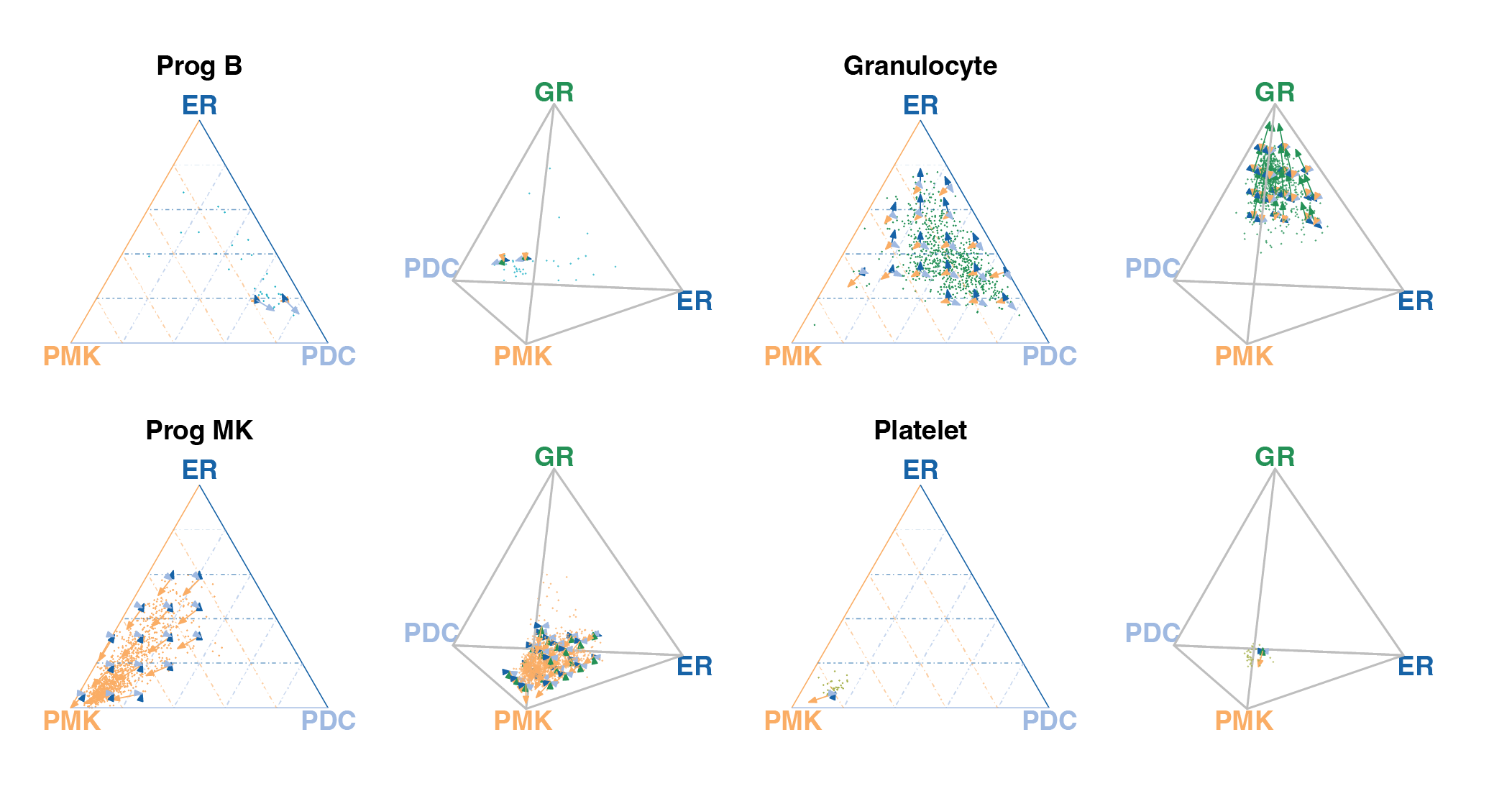
**Supplementary Figure S5. Simplex and velocity analyses of HSPC data.** Continuing from **Supplementary Figure 4**, this figure includes the remaining clusters from HSPC data. Blue arrow and axis: Erythrocyte cluster (ER). Yellow: Progenitor Megakaryocyte cluster (PMK). Light blue: Progenitor Dendritic cluster (PDC). Green: Granulocyte cluster (GR). Dot colors indicate clusters.


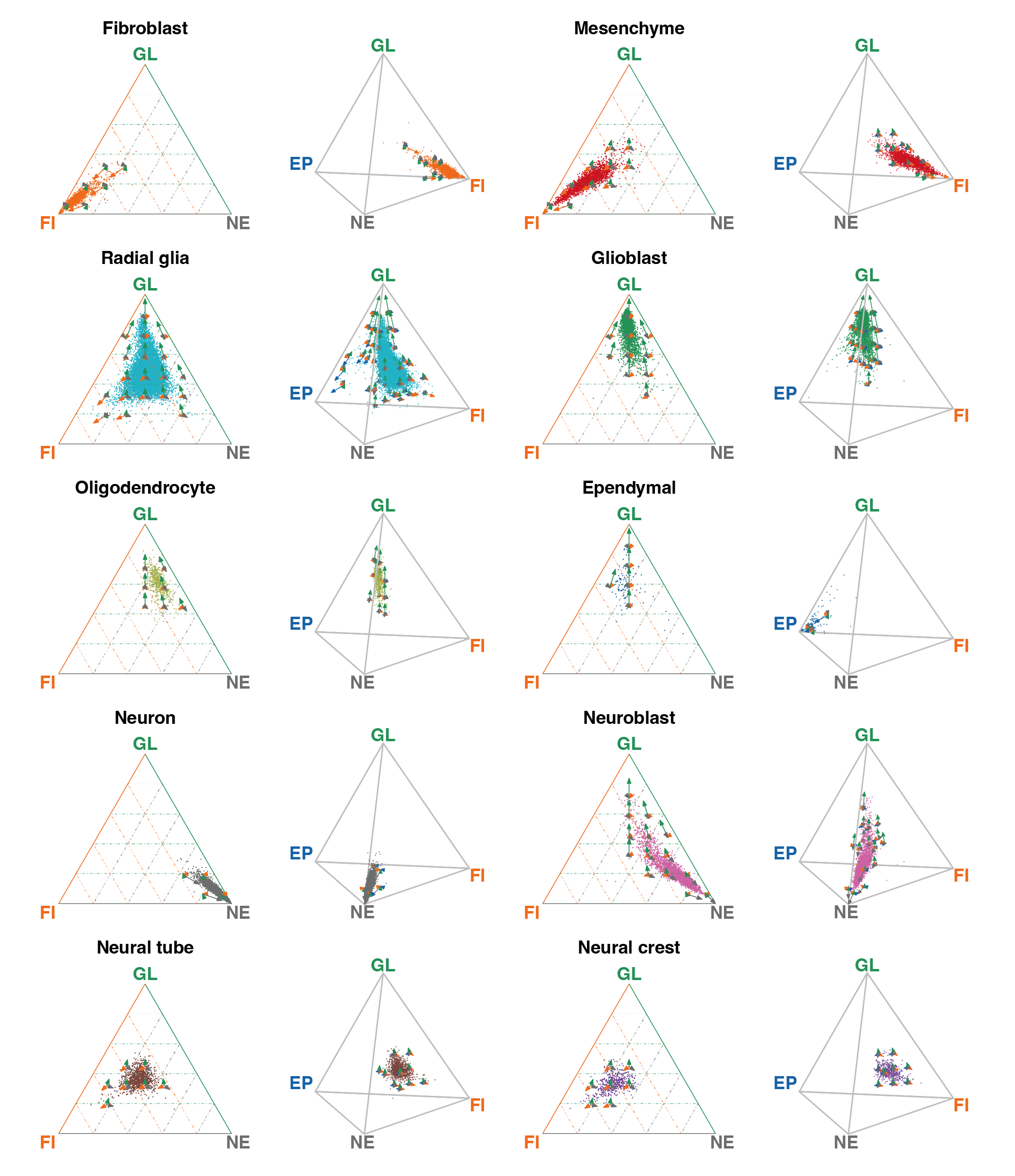
**Supplementary Figure S6. Simplex and velocity analyses of mouse brain atlas data.** Dots demonstrate the cell transcriptomic affinities towards three selected vertices, whereas the arrows show the future differentiation potential of the cells. Ternary simplex plots are shown in the first and third columns, while quaternary simplex plots are shown in the second and fourth columns. Green arrow and axis: Glioblast cluster (GL). Gray: Neuron cluster (NE). Orange: Fibroblast cluster (FI). Blue: Ependymal cluster (EP). Dot colors indicate clusters.

**
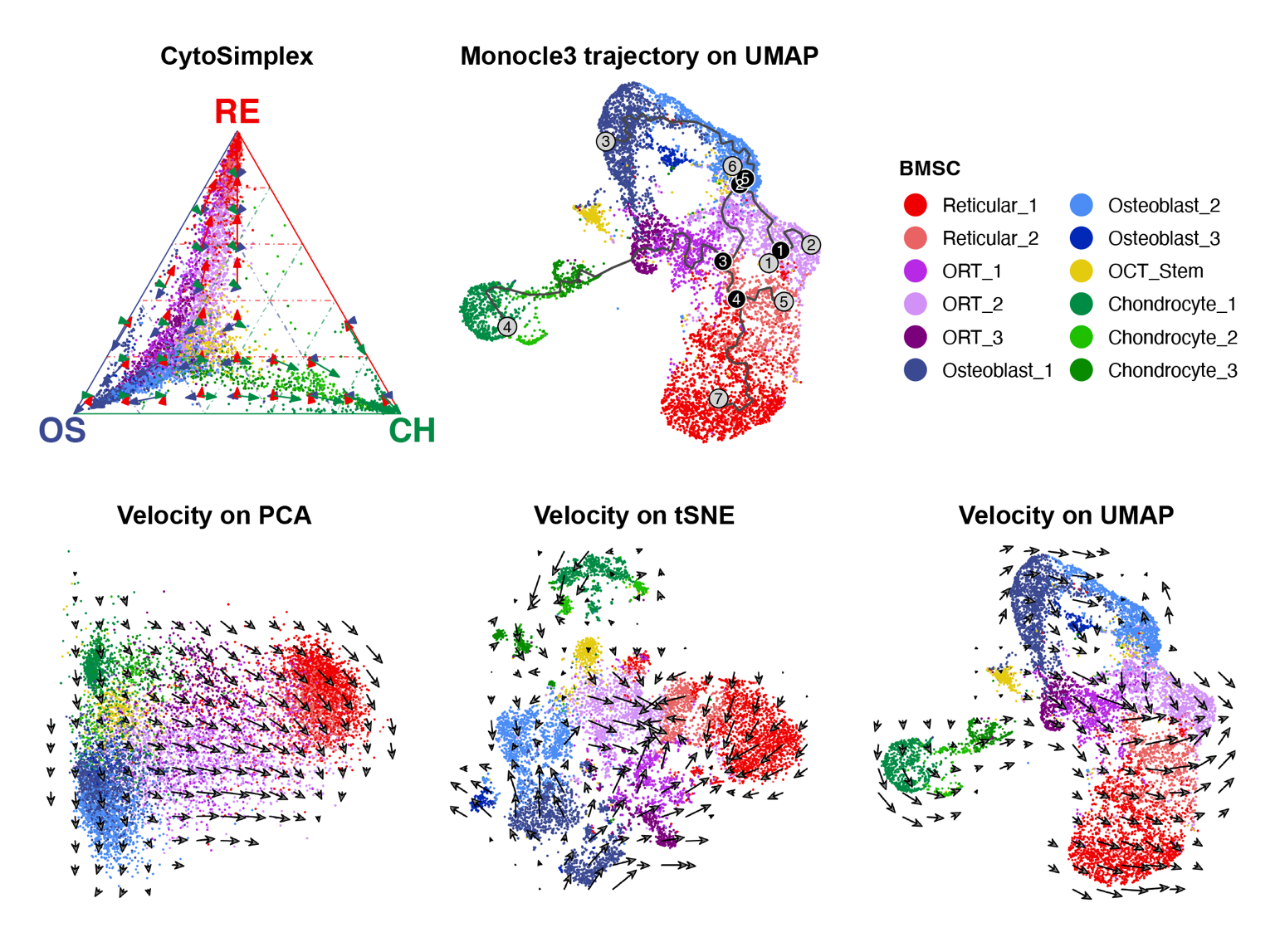
Supplementary Figure S7. Different approaches to visualize the cell fate of the BMSC dataset.**
