# Supplementary figures and images for: CytoSimplex: visualizing single-cell fates and transitions on a simplex
Source: Bioinformatics. 2025 Mar 22;41(4):btaf119. doi: 10.1093/bioinformatics/btaf119 (PMC11992338; doi:10.1093/bioinformatics/btaf119)

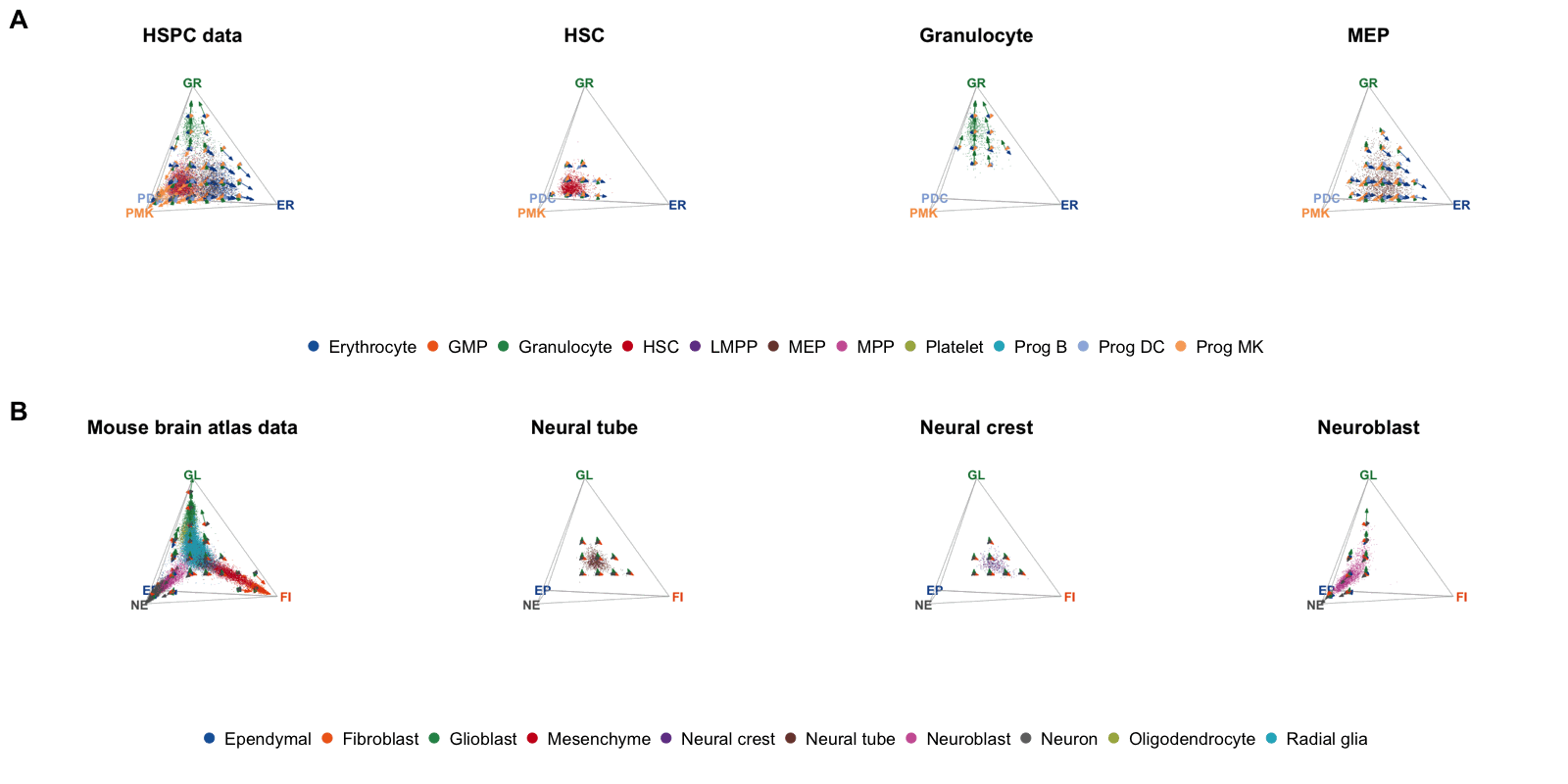

Supplement: btaf119_Supplementary_Data [file btaf119_supplementary_data.zip › Supplementary_Figure_S1.gif]
